# First case of *Trichinella spiralis* infection in beavers (*Castor fiber*) in Poland and Europe

**DOI:** 10.1016/j.ijppaw.2019.11.005

**Published:** 2019-12-05

**Authors:** Mirosław Różycki, Ewa Bilska – Zając, Maciej Kochanowski, Katarzyna Grądziel-Krukowska, Jolanta Zdybel, Jacek Karamon, Jan Wiśniewski, Tomasz Cencek

**Affiliations:** a Department of Parasitology and Invasive Diseases, National Veterinary Research Institute in Pulawy, Al. Partyzantow 57, 24-100, Pulawy, Poland; b Department of Food Hygiene and Public Health Protection, Faculty of Veterinary Medicine, Warsaw University of Life Sciences – SGGW, Nowoursynowska 159, 02-776, Warsaw, Poland

**Keywords:** *Castor fiber*, *Trichinella spiralis*, First case

## Abstract

**Background:**

This is the first report of the finding of *Trichinella spiralis* in beaver meat (*Castor fiber*) in Poland and Europe. In Poland, the beaver is a strictly protected animal species, except the few regions where high population density leads to economic losses. In these areas, the reduction culling of the animals was introduced. This uncommon hunting game animal is consumed and treated as a delicacy by hunters. However, currently, there is a lack of knowledge on possible risk factors for humans associated with the consumption of beaver meat. This paper presents the result of the study on the occurrence of nematodes of the genus *Trichinella* in beavers.

**Methods:**

In total, 69 beavers were examined for the presence of *Trichinella* spp. The 50g samples were taken from each animal and digested separately, according to a procedure based on the EU reference method. The larva DNA was examined by PCR and sequencing.

**Results:**

One of the 69 examined beavers was infected. Only one *Trichinella* larva was detected by the digestion method. The result of PCR confirms the presence of *T. spiralis* in beaver meat.

**Conclusions:**

This case further confirms the ability of these typical herbivores to be infected with *Trichinella* spp. This is the second confirmed case of *Trichinella* spp. infection in beavers in Europe and the first of *T. spiralis*.

## Highlights

• First report of the presence of *Trichinella spiralis* larva in beaver meat.
• Detection of *Trichinella spiralis* by digestion method and confirmed by PCR and sequencing.
• New parasitic risks factor for humans associated with consumption of beaver meat.

## Graphical abstract

**Figure:**
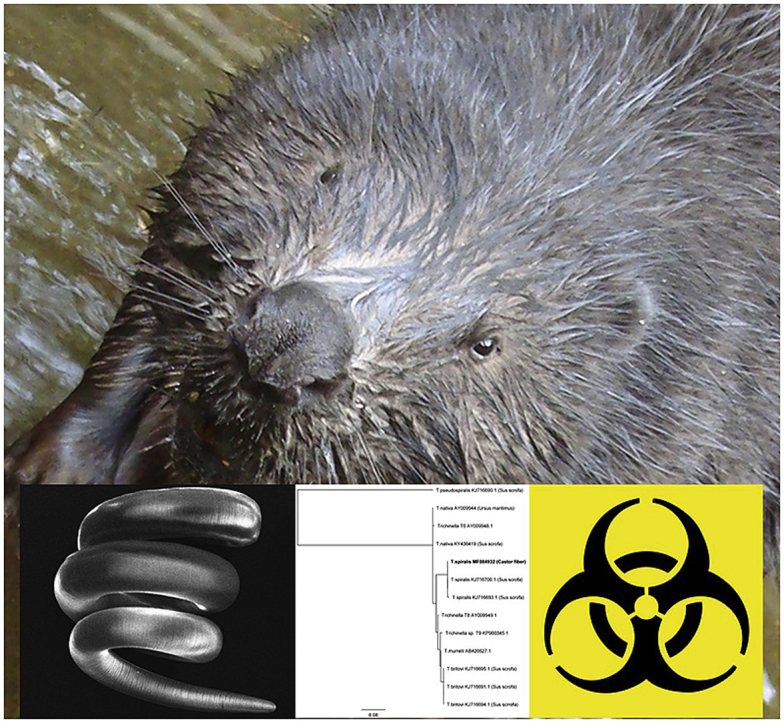

## 1 Introduction

Beavers are the largest rodents in Eurasia (Romanowski and Winczek, 2018). Adults are weighing 20–30 kg. In the past, they were very intensively hunted for fur and castoreum. In Poland, beaver meat was traditionally consumed by hunters and treated as a delicacy (Florek et al., 2017). In the Middle Ages, the beaver was considered as one of the most common animal species in Poland. For high-value furs, the privilege of beaver hunting was reserved for dukes only. Although in the Middle Ages beavers were under the protection of Polish kings, in the 13th century the number of beavers began to decrease. Intensive hunting led to the slow disappearance of beavers from the Polish territory. At the beginning of the 20th century, beavers became almost extinct (Biedrzycka et al., 2014). The species became strictly protected since 1919. The number of beavers was in slight growth, and the population was estimated at 400 animals in 1939. Beaver's protection contributes to the continuing increase of the population, which exceeds 120 000 in 2016 (http://stat.gov.pl/files/, 2017). Nowadays, beavers are protected, but their population increased to the level in which they can make serious damage in the rural environment. Therefore, in some places, the number of free living beavers has to be reduced (Misiukiewicz et al., 2018). The meat of hunted animals again becomes an object of consumption (Florek et al., 2017). In 2015, after the publication on the first findings of *Trichinella britovi* in beavers in Latvia, we decided to start research on this uncommon game animal (Seglina et al., 2015). The parasitic nematodes of genus *Trichinella* are infective to a wide range of hosts, mainly mammals but may also infect birds or reptiles (Pozio and Zarlenga, 2013). Parasite - host adaptation can be observed within the *Trichinella* genus (Pozio, 2007; Oivanen et al., 1645; Pozio, 2016). Four *Trichinella* species (*T. spiralis, T. britovi, T. nativa*, and *T. pseudospiralis*) occur in Poland (Bien et al., 2016a; Moskwa et al., 2012; Chmurzynska et al., 2013; Cabaj et al., 2000; Bilska-Zajac et al., 2017). Among game animals, only wild boars are routinely tested for *Trichinella*, thus there are no official data on examined beavers. The study was aimed to investigate the presence of *Trichinella* spp. in beavers hunted in Poland and assess the risk associated with the consumption of beaver's meat.

## 2 Materials and methods

Samples. In total 69 meat samples were obtained for the study. Samples (50g–100g) were taken from the rear left leg from *biceps femoris* and submitted voluntarily by hunters. The beavers were hunted within the frame of regulation shooting in the following regions: Subcarpathian region- 63 animals, Lesser Poland - 2, Pomerania - 2 and Masuria - 2. All samples were handled under the rules of the Commission Regulation (EU) No 749/2011 (https://publications.euro). **Digestion.** Meat samples weighing 50g were digested individually by the magnetic stirrer method (MSM) according to the reference method of Annex III Commission Implementing Regulation (EU) 2015/1375 (https://eur-lex.europa.eu, 2015; Mayer-Scholl et al., 2017). **Examination under Trichinoscope.** Digestion fluid was examined under Trichinoscope FFVII with 50x - 80× magnification. The collected larva was preserved in 96% ethyl alcohol for further DNA isolation. **DNA isolation.** he DNA was extracted and purified with the use of IQ™ System kit (Promega, USA). **Multiplex PCR.** The recognition of species was provided by the multiplex PCR method according to the protocol given by the European Reference Laboratory for Parasites (EURLP) (EURLP). PCR products were separated in a 2% agarose gel electrophoresis and stained with ethidium bromide. DNA bands in gel were visualized by exposure of the gel to ultraviolet light. The species was identified comparing the size of ITS1, ITS2 and ESV fragments produced by the amplification. As a positive control reference *Trichinella* larvae: *T. britovi, T. spiralis T. papaue* and *T. pseudospiralis, T. spiralis* were used. Nuclease-free water served as a negative control. **PCR and sequencing.** DNA extracted from larva was subjected to the PCR amplification of a fragment of part of 5S ribosomal DNA and fragment of mitochondrial cytochrome C oxidase 1 (CO1). The PCR for amplification of 5S rDNA and CO1 was performed according to previously described methods (Franssen et al., 2015). The PCR products were resolved by 2% agarose gel electrophoresis, stained with ethidium bromide and sizes determined in comparison with a standard 100bp ladder under UV. PCR products were purified using ExoSAP (Affymetrix, UK) according to manufacturer procedure. Sequencing was performed in thermal cycler Applied Biosystem Veriti. The obtained products were separated using genetic analyser (3730xl DNA Analyser, Applied Biosystems). Forward and reverse sequences were aligned and edited manually using the Geneious R7, ClustalW and Mega 5 (Kearse et al., 2012).

## 3 Results

**Fig. 1:**
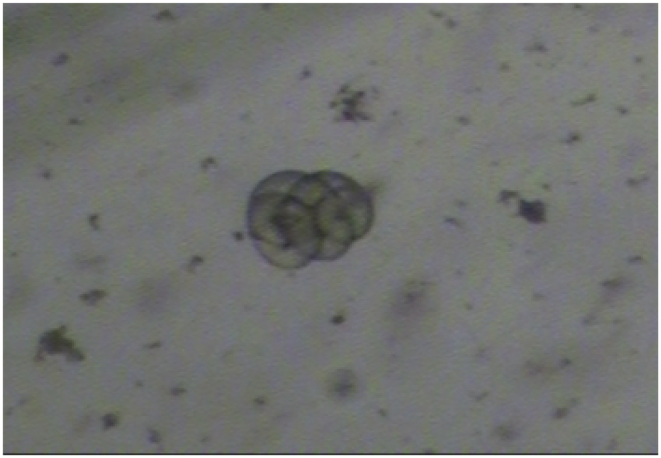
Larva digested from beaver muscle tissue morphologically identified as *Trichinella* spp. (magnification 120x), Trichotele VKC 1310.

**Fig. 2:**
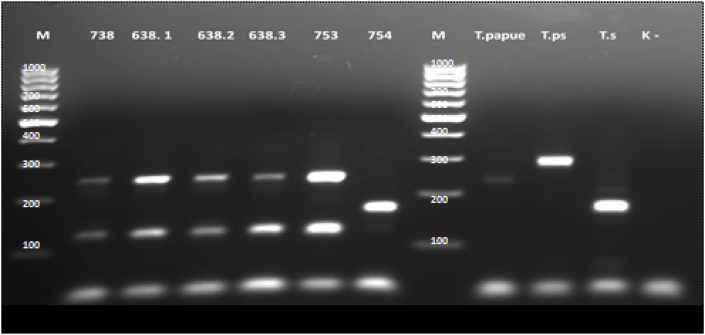
Electrophoretic profiles of *Trichinella* spp. larva amplicons after multiplex PCR amplification. Lanes 1 and 8 marker (Generuler 100 bp DNA), 2–6 (*T. britovi*), 7 - *T. spiralis* from beaver (sample 754), lanes 9–12 controls (9 - T. *papaue*, 10 - T. *pseudospiralis*, 11 - T. *spiralis*, 12 - negative control).

**Fig. 3:**
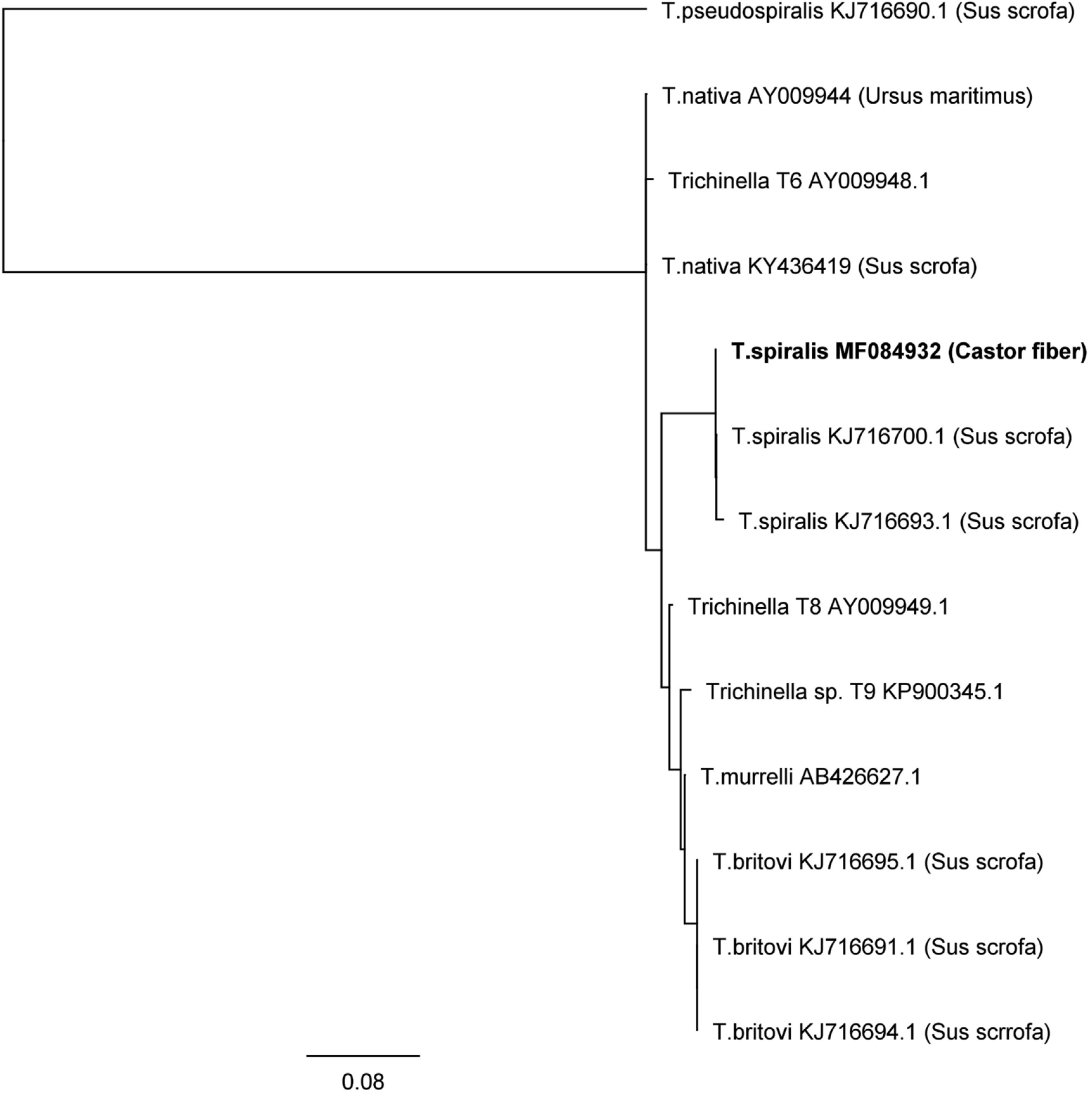
The Neighbor-Joining tree of Trichinella species inferred from 5S rDNA inter-gene spacer region sequences. Phylogeny Test - Bootstrap method, No. of Bootstrap Replications - 10000 Jukes Cantor model.

One of 69 examined beavers was found to be infected with *T. spiralis* (1,4%). The Regional Veterinary Laboratory in Krosno (Subcarpathian voivodeship) provided a positive sample. The density of invasion was 0.02 larva per gram (lpg). Based on the morphological characteristic larva was recognized as belonging to genus *Trichinella* (Fig. 1). The parasite was identified by mPCR as *T. spiralis* (Fig. 2). Forward and reverse sequences were aligned with Geneious R7 and checked using the freeware computer programs ClustalW and Mega 5. Results of alignments of amplified 5S rDNA sequences from the sample demonstrated 100% identity with sequences of *T. spiralis* originated from Polish wild boars (GeneBank access numbers KJ716696-KJ716745). Additionally, analysis of the alignment of CO1 sequence amplified from examined samples show 100% identity with reference sequence CO1 of *T. spiralis* (GeneBank access number KU321693 (Spiridonov et al., 2016), KM357422 (Mohandas et al., 2014). Generated phylogenetic tree clustered the analyzed sequences of *T. spiralis* from beaver in separated clad with other sequences of *T. spiralis* (Fig. 3). The analysis of both 5s rDNA and CO1 sequences from discovered larva confirms the findings. Obtained nucleotide sequences were deposited in GenBank under the following accession numbers MF084931 and MF084932.

## 4 Discussion

The research resulted in finding a single larva of the *T. spiralis* in the meat of the beaver hunted in Poland. This is also the first case of infection with *T. spiralis* in beaver in Europe. It has to be emphasized that there are only a little data on the *Trichinella* infection in beavers all over the world (Seglina et al., 2015; Bronstein and Lukashev, 2018; Rausch et al., 1956). The information presented by Raush on the presence of *T. spiralis* in Alaskan mammals in 1956 might be uncertain since the genetic methods were not involved at that time for species identification and only one species *T. spiralis* within genus *Trichinella* was described at those days. In Europe, *T. britovi* infection in beaver was detected for the first time in Latvia in 2015 - over 1000 km far from the Subcarpathian region 6. It has to be highlighted that dominative species in Latvia is *T. britovi.* In Poland, the dominative *Trichinella* species in fox population is *T. britovi* while in pigs and wild boars *T. spiralis* (Cabaj et al., 2000; Bilska-Zajac et al., 2013). The beaver was hunted in the region that borders with Bieszczady and Magura National Parks. The presence of *Trichinella* spp. in wildlife in this region was previously confirmed in numerous publications (Bilska-Zajac et al., 2013; Cybulska et al., 2016; Moskwa et al., 2013; Bien et al., 2016b). Data collected by the National Reference Laboratory (NRL) showed that the prevalence of *Trichinella* spp in the wild boar population in this region is 0.23% with *T. spiralis* to *T. britovi* ratio of 3:1 ^26,29^. However, surveillance study on *Trichinella* species in red foxes indicates *T. britovi* as predominant species for this host and region, the same as in Slovakia (Hurnikova and Dubinsky, 2009). The origin of *Trichinella* infection in beavers is unknown since these animals are typical herbivores (Florek et al., 2017; Gorczyca et al., 2018). In spite of this, in hard conditions animals for supplementation of protein or mineral deficits may feed on things they don't usually include in their diet. Incidental consumption of feed contaminated with infected meat or infection during a fight with predators invading beaver settlements can't be excluded 6.

## 5 Conclusions

The first detection of *T. spiralis* in European beaver in Poland is the next evidence confirming the ability of herbivores to be the unspecific host for *Trichinella* nematodes. Does it mean that we have a new source of infection or just a single case without an important outcome to risk? The number of examined animals is too low to make a final statement that this herbivore, is a newly established vector for *Trichinella*. Taking into account the little worldwide data on *Trichinella* infected beavers, in our opinion, it is an accidental case and only further research may give us an answer. The number of hunting permits rises in hunting season 2019–2020 to nearly 3000, thus we expect more epidemiological data soon.

## Animal right statement

The study was conducted on legally hunted animals, all samples were handled under the rules of the Commission Regulation (EU) No 749/2011.

## Author contributions

M. R. - main idea and work organization.

E. B-Z. and M.K. carried out the molecular study.

K. G-K. and J. Z. performed a magnetic stirrer digestion method.

J. K. and J. W. - sample collection from hunters, transport samples to laboratory and contribution to the final version.

T. C. - helped supervise the project.

## Declaration of competing interest

The authors declare that there is no conflict of interest regarding the publication of this article.
